# The prevention and treatment of COVID-19 in patients treated with hemodialysis

**DOI:** 10.1186/s40001-023-01389-9

**Published:** 2023-10-09

**Authors:** Binyu Zeng, Jia Zhou, Daizhuang Peng, Chengmei Dong, Qun Qin

**Affiliations:** 1 National Institution of Drug Clinical Trial, Xiangya Hospital, Central South University, Changsha, 410008 China; 2 National Clinical Research Center for Geriatric Disorders, Xiangya Hospital, Central South University, Changsha, 410008 China; 3 International Science and Technology Innovation Cooperation Base for Early Clinical Trials of Biological Agents in Hunan Province, Changsha, China

**Keywords:** Hemodialysis, Anti-COVID-19 drug, COVID-19 vaccine, Safety, Efficacy

## Abstract

Patients treated with hemodialysis are often immunocompromised due to concomitant disease. As a result, this population is at high risk of infection and mortality from COVID-19. In addition to symptomatic treatment, a series of antiviral drugs targeting COVID-19 are now emerging. However, these antivirals are used mainly in mild or moderate patients with high-risk factors for progression to severe disease and are not available as pre- or post-exposure prophylaxis for COVID-19. There is a lack of clinical data on the use of anti-COVID-19 drugs, especially in patients treated with hemodialysis, therefore, vaccination remains the main measure to prevent SARS-CoV-2 infection in these patients. Here, we review the clinical features and prognosis of patients on hemodialysis infected with SARS-CoV-2, the main anti-COVID-19 drugs currently available for clinical use, and the safety and efficacy of anti-COVID-19 drugs or COVID-19 vaccination in patients treated with hemodialysis. This information will provide a reference for the treatment and vaccination of COVID-19 in patients treated with hemodialysis and maximize the health benefits of these patients during the outbreak.

## Introduction

Since the beginning of 2020, a new coronavirus has swept the world, posing a great threat to the health and safety of people all over the globe. The World Health Organization declared the outbreak a global pandemic in May 2020 and named the infection caused by the SARS-CoV-2 virus Corona Virus Disease 2019 (COVID-19). Similar to Middle East Respiratory Syndrome (MERS) and Severe Acute Respiratory Syndrome Coronavirus (SARS), COVID-19 mainly impacts the respiratory system, with common clinical manifestations, including cough, dyspnea, fever, and sore throat [1–4]. However, COVID-19 has also been shown to affect other organs and tissues, and its extra-pulmonary manifestations include the cardiovascular system, kidney, liver, gastrointestinal, eye, skin and nervous system [5, 6]. As the pandemic developed, research and development of COVID-19 vaccines and therapeutic drugs was quickly initiated in countries around the world. The pandemic situation remains serious, and in addition to maintaining good hygiene practices, the implementation of strict protective measures is still highly important.

At present, COVID-19 is managed mainly by symptomatic treatment, anti-COVID-19 drugs and vaccination. To date, several anti-COVID-19 drugs have been approved for emergency use, but these drugs are primarily used in mild or moderate patients with high-risk factors for progression to severe disease and are not available as pre- or post-exposure prophylaxis for COVID-19. Emergency access to several COVID-19 vaccines has also been granted in multiple countries. Up to now, the main types of COVID-19 vaccines include inactivated vaccines, non-replicating virus vector vaccines, RNA vaccines, DNA vaccines, protein subunit vaccines and virus-like particle (VLP) vaccines.

Hemodialysis is the main treatment for patients with kidney failure, and patients treated with hemodialysis have immune deficiency due to T lymphocyte proliferation and transplantation, decreased IL-2 production and abnormal levels of other cytokines, as well as bacterial lipopolysaccharide contamination of dialysate, which can also mediate immune dysfunction [7]. Therefore, this group of patients will face a high rate of SARS-CoV-2 infection and mortality, as well as the risk of ineffective vaccination. Clinical data on the use of anti-COVID-19 drugs in patients treated with hemodialysis at this stage are limited, and vaccination remains the primary method of the prevention and treatment of disease in this population. The Technical Guidelines for COVID-19 Vaccination (First Edition) issued by the National Health Commission of the People’s Republic of China defines immunocompromised people as one of the specific populations eligible for COVID-19 vaccination. However, due to immune dysfunction, patients treated with hemodialysis are often excluded from initial clinical studies of COVID-19 vaccines. However, several studies worldwide have evaluated the safety and efficacy of COVID-19 vaccines, mainly mRNA vaccines and adenoviral vector vaccines, in this population [8–10]. Here, we review the main clinical anti-COVID-19 drugs available at this stage, and the safety and efficacy of anti-COVID-19 drugs or COVID-19 vaccination in patients treated with hemodialysis. This information will serve as a reference for the prevention and treatment of COVID-19 in this population to maximize the health benefits of patients treated with hemodialysis during the pandemic.

## Clinical characteristics and prognosis of patients on hemodialysis with COVID-19

Previous studies have shown that the delivered dose of hemodialysis therapy is an important predictor of patient mortality. The mortality decreased with an increase in the delivered dose of hemodialysis represented by the urea kinetic modelling parameter Kt/V(K = dialyzer clearance, *t* = dialysis time, *V* = volume of distribution of urea) [11, 12]. The main reasons for inadequate hemodialysis include insufficient dialysis time, poor permeability of dialyzer, and vascular access recirculation [13]. To maintain a normal life, patients with kidney failure must attend hospital to undergo hemodialysis 3–4 times a week, with each session lasting 3–5 h [14]. In this special period, this will inevitably increase the risk of patients infected with SAR-CoV-2, and will also affect patient compliance and lead to inadequate hemodialysis. Vascular access is considered the lifeline for patients on hemodialysis, throughout the pandemic, many patients on hemodialysis have not received timely intervention for vascular access care [15]. Therefore, patients treated with hemodialysis have a high risk of SAR-CoV-2 infection and a high mortality than the general healthy population. In a study of a European kidney replacement therapy population, Jager et al. reported that the attributable mortality rate for COVID-19 was 20.0% in 3285 patients on dialysis after a 28-day follow-up [16]. In a survey of nasopharyngeal swab tests for COVID-19 obtained from 1722 patients treated with hemodialysis in a region of northern Italy, Rombolà et al. reported that 553 (32.1%) tested positive and the death was 171, representing a case fatality rate of 30.9% and mortality was 3.2%, which was much higher than the corresponding rates in the general population [17]. The main clinical symptoms in patients on hemodialysis with COVID-19 are fever, cough, fatigue and dyspnea, while some patients are asymptomatic [18, 19]. It is also accompanied by abnormal laboratory results, such as lymphopenia, anemia and elevated C-reactive protein [20].

Mortality is higher in patients on hemodialysis with COVID-19 compared to those with COVID-19 who did not require hemodialysis [21]. Relevant studies have shown that old age, male and chronic diseases are the main risk factors for high mortality in this group [16, 19]. The sex difference in the mortality rate among COVID-19 patients on dialysis is consistent with previous studies demonstrating a similar phenomenon sex as a recognized feature of chronic progressive kidney disease and that differences in the hormonal environment make being female a protective factor for this condition [22–24]. In addition, female sex hormones play an important role in immune protection against infection, which may account for the significantly lower prevalence of COVID-19 in women than in men [25]. In addition, COVID-19 can cause further kidney injury, which may exacerbate pre-existing kidney disease in patients treated with hemodialysis, leading to increased mortality [26].

## Anti-COVID-19 drugs authorized for emergency use

### Convalescent plasma

Convalescent plasma, which has a long history of use in infectious diseases, involves the delivery of a certain titer of specific antibodies and other immune components present in the plasma isolated from convalescent patients to treat the disease. It can be used to quickly identify and capture the virus, activate the complement system, thereby eliminating the virus. Many existing randomized clinical trials have demonstrated good efficacy of convalescent plasma against infectious diseases caused by viruses, such as SARS [27], influenza A virus (H1N1) [28], animal influenza A virus (H5N1) [29] and Ebola virus [30]. Available clinical data indicate that high-titer convalescent plasma is only moderately effective for reducing the risk of developing severe COVID-19 in older patients with mild infection, but the results are not statistically significant [31]. For people with moderate or severe SARS-CoV-2 infection, the efficacy appears to be even less promising. A randomized, multicenter and single-blind clinical trial exploring the effectiveness of high-titer convalescent plasma administered to high-risk outpatients within 1 week after the onset of COVID-19 symptoms showed that disease progression occurred in 77 (30.0%) patients in the convalescent plasma group and 81 (31.9%) patients in the placebo group. Furthermore, five patients died in the convalescent plasma group and one patient in the placebo group. These data indicated that the administration of COVID-19 convalescent plasma to high-risk outpatients did not prevent disease progression [32]. A randomized controlled trial conducted in adult patients hospitalized with COVID-19 in the United States showed that high-titer convalescent plasma did not improve survival or other pre-specified clinical outcomes after delivery at any stage [33], a finding that has been supported by data from numerous other clinical trials [34–36].

### Intravenous immunoglobulin

Intravenous immunoglobulin (IVIG) is a blood product containing IgG antibodies isolated from a healthy donor, which have immunomodulatory and immune replacement effects. This therapy has a wide range of applications in a variety of inflammatory, infectious, autoimmune and viral diseases [37]. Data from numerous clinical studies show that IVIG improves clinical outcomes and reduces mortality in early stage COVID-19 patients [38, 39], with shorter hospital stays and reduces duration of mechanical ventilation [40]. However, IVIG does not seem to produce satisfactory results in patients with late stage COVID-19 and moderate or severe infection. A meta-analysis showed that IVIG treatment had no significant impact on mortality or length of hospital stay in adult COVID-19 patients, therefore, IVIG not recommended for this population [41]. Another multicenter, double-blind, placebo-controlled phase III trial showed that IVIG did not improve clinical outcomes in COVID-19 patients receiving mechanical ventilation with moderate-to-severe acute respiratory distress syndrome, and appeared likely to lead to an increased frequency of serious adverse events [42]. In addition, a randomized controlled clinical trial suggested that IVIG in combination with standard therapy was safe and effective in patients with COVID-19-associated moderate pneumonia [43]. Therefore, it is recommended that IVIG be used early in patients with COVID-19 or in combination with other antiviral agents for the treatment of COVID-19.

### Monoclonal antibodies

Monoclonal antibodies (mAbs) are produced by artificially prepared hybridoma cells, which are formed by the fusion of sensitized B cells that secrete specific antibodies with myeloma cells that have infinite reproduction capacity. Compared with polyclonal antibodies, monoclonal antibodies have the advantages of high purity, high sensitivity and strong specificity, although the cost of production is higher. The emergence of mAbs has provided new opportunities for the treatment of human diseases and mAbs are now widely used in the treatment of cancer and autoimmune diseases as well as inflammatory and infectious diseases. In addition, due to their high specificity and sensitivity, mAbs can also be used as a powerful tool for the detection and treatment of many diseases [44]. The SARS-CoV-2 S glycoprotein consists of the S1 subunit and the transmembrane S2 subunit. The S1 subunit contains the N-terminal domain (NTD) and the receptor-binding domain (RBD), and neutralizing antibodies targeting the RBD, NTD and S2 have been reported to be potentially translatable for human use [45]. Over time, SARS-CoV-2 mutates to generate new types, making it necessary to identify new mAbs that recognize these to new strains or to adopt a combination of mAbs to avoid virus escape. Worldwide, a number of mAbs have shown promising therapeutic and prophylactic effects against SARS-CoV-2 without significant safety concerns. The main mAbs that have been authorized for emergency use, such as REGN‑COV2 (casirivimab + imdevimab) [46], LY-CoV555 + LY-CoV016 (bamlanivimab + etesevimab) [47] and AZD7442 (tixagevimab + cilgavimab) [48].

### Paxlovid (nirmatrelvir + ritonavir)

Nirmatrelvir + ritonavir (trade name Paxlovid), which was developed by Pfizer, is an oral small molecule COVID-19 therapeutic drug. Nirmatrelvir is an oral antiviral agent that targets the SARS-CoV-2 viral main proteinase (Mpro, also known as 3CLpro), thereby inhibiting viral replication, but has the disadvantage of being susceptible to metabolism by the hepatic enzyme CYP 3A4. The pharmacokinetics of Nirmatrelvir are enhanced by combination with the CYP 3A4 inhibitor, Ritonavir [49]. Therefore, Paxlovid should not be used in combination with strong inducers of CYP3A4, drugs that are highly dependent on CYP3A4-mediated clearance and those for which elevated plasma concentrations may lead to serious or life-threatening adverse reactions. Thus, numerous drug interactions may limit the widespread use of Paxlovid. Such COVID-19 drug interactions can be predicted by searches of a new database (https://www.covid19-druginteractions.org/) that is freely available. A phase II/III, double-blind, randomized, controlled EPIC-HR trial demonstrated that treatment of adult COVID-19 patients with high-risk factors for progression to severe disease with nirmatrelvir (300 mg) + ritonavir (100 mg) every 12 h for 5 days, resulted in 89% lower risk of COVID-19-related hospitalization or death from any cause compared with the placebo group, and without significant safety concerns [50]. In addition, a real-world observational study of the clinical effectiveness of nirmatrelvir + ritonavir in community-dwelling outpatients with COVID-19 in Hong Kong showed that early application of this combination reduced mortality, risk of in-hospital disease progression and risk of hospitalization in this population [51].

### Molnupiravir

As the first oral anti-COVID-19 drug developed in the world, Molnupiravir is a small molecule ribonucleoside prodrug of N-hydroxycytidine (NHC), which is absorbed by cells and phosphorylated to form active ribonucleoside triphosphate (NHC-TP). This product is then incorporated into the RNA of SARS-CoV-2 via viral RNA polymerase, causing mis-replication of the viral genome and ultimately, a non-infectious virus [52, 53]. However, the ability of Molnupiravir to induce mis-replication of viral RNA is associated with an increased risk of mutation in human cells. Thus, the use of Molnupiravir in pregnant women and children should be restricted. Due to the lack of clinical data, interactions of Molnupiravir with other clinical agents have not been identified. Interim staging results from a phase III randomized controlled placebo clinical trial exploring the efficacy and safety of Molnupiravir in ambulatory COVID-19 patients revealed a significantly lower rate of hospitalization or mortality in the Molnupiravir group compared with the placebo group (7.3% vs.14.1%, *P* = 0.001). These findings indicated that, in high risk and unvaccinated adult patients with COVID-19, early treatment with Molnupiravir was effective in reducing the risk of hospitalization and mortality. However, the treatment was found to be less effective in a fully randomized study population, with lower rates of hospitalization or mortality in the Molnupiravir group and placebo groups (6.8% vs. 9.7%) [54].

### VV116

Remdesivir was previously approved for marketing as an anti-COVID-19 drug. However, the WHO issued a statement that Remdesivir was not recommended for COVID-19 treatment because of its limited therapeutic effect and no evidence that the drug improves patient survival or reduces the need for ventilation [55]. VV116 is a deuterated tri-isobutyrate prodrug of the Remdesivir parent nucleoside and is rapidly metabolized into the parent nucleoside (116-N1) in vivo. 116-N1 is then converted intracellularly to the nucleoside triphosphate active form, thereby interfering with the function of RNA-dependent RNA polymerase of COVID-19 and exerting antiviral effects [56]. VV116 was developed as an oral, small molecule anti-COVID-19 drug for adult patients with mild-to-moderate COVID-19 and was conditionally approved for marketing in China on January 29, 2023. An open, prospective cohort study showed a shorter time to viral shedding in subjects treated with VV116 within 5 days of the first positive test for COVID-19 than in controls [57]. In addition, a phase III, non-inferiority, observer-blinded randomized clinical trial comparing VV116 with Nirmatrelvir + Ritonavir in adult patients with mild to moderate COVID-19 and high-risk factors for progression to severe disease showed that VV116 was non-inferior to Nirmatrelvir + Ritonavir in the duration of clinical recovery and had fewer safety concerns [58].

### Azvudine

Azvudine was the first dual-targeted nucleoside antiviral drug to be developed, and was previously used primarily for the prevention and treatment of HIV infection. In addition to inhibiting HIV-1 reverse transcription, Azvudine also restores cytidine deaminase APOBEC3G (A3G) expression in CD4^+^ T cells from Azvudine-treated HIV-1 patients by binding to the Vif-E3 ubiquitin ligase complex and inhibiting Vif-induced ubiquitination and degradation of A3G, thereby preventing HIV-1 replication [59]. Preliminary results from a small randomized, open-label, controlled clinical trial of 20 subjects exploring the efficacy and safety of Azvudine for COVID-19 showed an average time to first nucleic acid negative conversion of 2.60 days in the Azvudine group compared with 5.60 days in the control group. On this basis, it was hypothesized that Azvudine treatment of patients with mild and common COVID-19 may shorten the time to first nucleic acid negative conversion compared to standard antiviral therapy, although a larger sample size is needed to verify this conclusion [60]. In terms of the therapeutic mechanism, it has been postulated that the Azvudine triphosphate embeds in, and inhibits related polymerases during SARS-CoV-2 RNA synthesis, ultimately leading to the termination of RNA replication [61]. The National Medical Products Administration of China has registered an application for emergency conditional approval of Azvudine for the treatment of indications of COVID-19.

### Metformin

As a pleiotropic antidiabetic agent, metformin has hypoglycemic effects and can also be widely used in cancer, inflammation, obesity, osteoporosis, periodontitis, polycystic ovary syndrome, aging, cardiovascular and neurodegenerative diseases [62, 63]. Many clinical studies have shown that metformin can reduce the severity and mortality of COVID-19 [64–66]. Studies suggested that the mechanism of metformin against COVID-19 may be related to anti-inflammatory, antiviral activity, the prevention of hyperglycemia during acute illness, reduction of endothelial injury and pulmonary fibrosis [67]. A phase III, randomized, double-blind, placebo-controlled trial conducted at six institutions in the United States, tested the efficacy of metformin, ivermectin, and fluvoxamine for early outpatients treatment of SARS-CoV-2 infection. The results showed that metformin reduced the incidence of emergency department visit, hospitalization, or death [68]. After that the study further evaluated whether outpatients with COVID-19 treatment with metformin, ivermectin, or fluvoxamine soon after SARS-CoV-2 infection could reduce the risk of post-COVID-19 condition, also known as long COVID [69]. The results suggested that outpatients with COVID-19 treatment with metformin reduced long COVID incidence by about 41%, compared with placebo. However, the results did not indicate whether metformin would be effective at preventing long COVID if started at the time of emergency department visit or hospitalization for COVID-19, or whether metformin would be effective as a treatment in people who already have long COVID. Due to the risk of hypoxemia, acute kidney disease, cardiovascular complications and acidosis, it is appropriate to consider withdrawing metformin when COVID-19 becomes more severe [70].

## Advantages and disadvantages of anti-COVID-19 drugs authorized for emergency use

Biological agents such as convalescent plasma, IVIG and mAbs are mostly administered by injection to achieve anti-COVID-19 effects by enhancing IgG antibody titers in patients. Compared with these biological agents, oral anti-COVID-19 chemical drugs have the advantages of stability, ease of administration and high patient compliance. According to the existing clinical data, the common adverse events of the commonly used anti-COVID-19 drugs mentioned above are mainly mild or moderate, and the incidence of serious adverse events is relatively low, supporting the good safety of all of these agents. Details of the safety of commonly used anti-COVID-19 drugs are shown in Table 1. To date, clinical data on the use of these anti-COVID-19 drugs in patients treated with hemodialysis are limited, making it difficult to make accurate judgments about their safety and efficacy in these patients. In response to the vulnerability to COVID-19 faced by immunocompromised patients, a real-life study in Chinese patients with COVID-19 calls for the implementation of early Paxlovid therapy in immunocompromised high-risk patients, including hospitalized patients and especially in unvaccinated patients, to promote viral eradication [71]. Another small-scale retrospective cohort study exploring the safety and efficacy of outpatient use of Molnupiravir in patients on hemodialysis with COVID-19 showed that Molnupiravir appeared to be safe and effective as an outpatient treatment for COVID-19 in patients treated with hemodialysis [72]. Future large-scale clinical studies are still needed to further investigate and confirm these preliminary results, and therefore, COVID-19 vaccination remains the best strategy to prevent COVID-19 in patients treated with hemodialysis.

**Table 1:** Safety of commonly used anti-COVID-19 drugs

Medicines	Cases (n)	AE (%)	SAE (%)	Common AE	Identifier
Convalescent plasma	468	NA	NA	Bleeding, Venous thromboembolism, Arterial thromboembolism	NCT04364737 [35]
Intravenous immunoglobulin	68	75.0%	32.0%	Abdominal or back pain, fever, headache, chills, rash, fatigue, nausea or vomiting	NCT04350580 [42]
REGN-COV2	3688	7.1–8.4%	1.1–1.7%	Infusion-related reactions and hypersensitivity reactions	NCT04425629 [46]
LY-CoV555 + LY-CoV016	518	13.3%	1.4%	Nausea, rash, dizziness, diarrhea and hypertension	NCT04427501 [47]
AZD7442	3461	35.3%	1.4%	Injection site reactions	NCT04625725 [48]
Paxlovid	1109	22.6%	1.6%	Dysphagia, diarrhea and vomiting	NCT04960202 [50]
Molnupiravir	710	30.4%	6.9%	Diarrhea, malignancy and dizziness	NCT04575597 [54]
VV116	384	67.4%	0.3%	Dysgeusia, hypertriglyceridemia and hyperlipidemia	NCT05341609 [58]
Azvudine	10	0.0%	0.0%	NA	ChiCTR2000029853 [60]
Metformin	663	NA	NA	Rhinorrhea, cough, fatigue, myalgia and headache	NCT04510194 [68]

*NA* not available, *AE* adverse event, *SAE* serious adverse event, *NCT* national clinical trial, *ChiCTR* Chinese clinical trial register

## Safety of COVID-19 vaccination in patients treated with hemodialysis

Vaccine safety is a primary concern, especially for patients on hemodialysis with severe underlying disease and immunodeficiency. French and Italian studies showed that concerns about adverse effects and efficacy of COVID-19 vaccines in dialysis patients were independent predictors of reluctance to receive the vaccine in this population [73]. Many studies have demonstrated the safety of COVID-19 vaccination in general populations, with local and systemic adverse reactions, including transient mild-to-moderate pain at the vaccination site, fatigue and headache, and a low incidence of serious adverse reactions [74–77]. However, several previous reports of rare but severe thromboembolism and thrombocytopenia following administration of ChAdOx1 nCov-19, a recombinant adenoviral vector vaccine developed by AstraZeneca, have led to renewed anxiety about the safety of COVID-19 vaccines [78].

To allay concerns, studies about the safety of the COVID-19 vaccine in patients treated with hemodialysis have been conducted. In a comparison of the incidence of side-effects of the mRNA COVID-19 vaccine in patients treated with hemodialysis and patients treated with peritoneal dialysis, Stumpf et al. found that patients treated with peritoneal dialysis had a higher incidence of vaccination-related side-effects, such as fever and vaccination site pain, than patients treated with hemodialysis. The authors speculated that this difference may be related to the fact that patients treated with hemodialysis have become accustomed to repetitive puncture pain and chronic microinflammation due to blood membrane contact, whereas patients treated with peritoneal dialysis are not [79]. Zitt et al. evaluated the safety of the BNT162b2 mRNA vaccine in 50 patients on hemodialysis with a mean age of 67.6 years. Mild and moderate site pain at the injection site was the most common local adverse effect, while systemic adverse effects were mainly diarrhea, fatigue, chills, muscle pain and arthralgia, although these were mostly mild. The authors also explored the relationship between local reactions and antibody response after completing the vaccination course, and although antibody titer was higher in patients with pain at the vaccination site than in patients without, the difference was not statistically significant. In addition, this study showed that despite intramuscular injections during hemodialysis, no significant local hematomas occurred due to the use of low molecular weight heparin anticoagulation [80]. Existing studies about the safety of COVID-19 vaccine in patients on hemodialysis have focused on inactivated vaccine and mRNA vaccine. To further compare the safety of inactivated vaccine and mRNA vaccine in patients treated with hemodialysis, researchers in Turkey conducted a clinical study comparing the side effects of inactivated vaccine (CoronaVac) and mRNA vaccine (BNT162b2) in this group. The results suggested that systemic adverse effects such as fever, malaise or generalized muscular pain were more often with BNT162b2 than CoronaVac (54% vs. 24%; *P* = 0.0049), but did not report any serious adverse events [81]. All of these studies provide evidence that the COVID-19 vaccine is well-tolerated by patients treated with hemodialysis. More details of the Clinical researches related to the safety of COVID-19 vaccination in patients treated with hemodialysis are shown in Table 2.

**Table 2:** Clinical researches related to the safety and efficacy of COVID-19 vaccination in patients treated with hemodialysis

Author, years	PHD (n)	Control (n)	Intervention	Safety outcome	Efficacy outcome
Goupil et al.[82], 2021	154	• Healthy control (40) • Convalescent plasma (16)	One dose of BNT162b2	NA	• A single dose of BNT162b2 vaccine failed to elicit a humoral immune response in most PHD without previous SARS-CoV-2 infection • The antibody response was delayed in PHD with previous SARS-CoV-2 infection
Zitt et al.[80], 2021	50	NA	Two doses of BNT162b2	• Most common local reactions: pain at the injection site (38% and 29.2% after the first and second injection, respectively) • Most common systemic reactive events: diarrhea (4% mild, 4% moderate) and fatigue (8% mild)	• Higher age and immunosuppression were associated with lower, calcitriol treatment and prior seroconversion to hepatitis B vaccination with significantly higher antibody concentration • A high immunogenicity in PHD
Jahn et al. [83], 2021	72	Healthy control (16)	Two doses of BNT162b2	NA	• PHD under 60 years of age responded equally to healthy controls • The antibody response in PHD negatively correlated with age
Speer et al. [93], 2021	22	Healthy control (46)	Two doses of BNT162b2	NA	• A reduced antibody response to the first and second doses of BNT162b2 in PHD • The majority (82%) develop neutralizing antibodies after the second dose but at lower levels than healthy controls
Raja et al. [100], 2022	72	Healthy control (72)	One dose of ChAdOx1 nCoV-19	NA	• A weak antibody response to the ChAdOx1 nCoV-19 in PHD • Older age was associated with no responders
Agur et al. [101], 2022	80	Healthy control (56)	Three doses of BNT162b2	NA	• A third dose of BNT162b2 substantially improved humoral response in PHD • Baseline antibody level, dialysis therapy, and hypoalbuminemia were independent predictors of impaired antibody response
Dheir et al. [91], 2022	50	• Kidney transplant recipients (64) • Healthy control (41)	Two doses of CoronaVac	NA	• A negative correlation between antibody seroconversion and age • The antibody response in PHD is almost comparable to healthy controls but kidney transplant patients have a poor response
Melin et al. [96], 2022	50	NA	Two doses of BNT162b2	NA	• A third dose of BNT162b2 gives a robust and improved immunological response in PHD
Espi et al. [92], 2022	106	Healthy control (30)	Two doses of BNT162B2	NA	• Impaired humoral and cellular immune responses to SARS-CoV-2 mRNA vaccine in virus-unexposed PHD
Gonzalez-Perez et al. [9], 2022	39	Healthy control (92)	• Patients (two doses of mRNA-1273) • Control (two doses of BNT162b2)	NA	• PHD develop strong cellular and humoral immune responses to 1273-mRNA vaccination
Stumpf et al. [79], 2022	1168	PPD (58)	Two doses of mRNA-1273 or BNT162B2	• Vaccination-related clinical side effects (fever and arm pain) occurred more often in PPD than in PHD	• No difference in COVID-19 mRNA vaccination-related adaptive immune responses in PPD and PHD
Puspitasari et al. [102], 2023	38	NA	Two doses of the CoronaVac and booster dose of BNT162b2	• Most common AE: mild pain at the injection site (55.26%), mild fatigue (10.53%), and swelling at the injection site (10.53%) • No SAE	• A good antibody response to the BNT162b2 booster vaccination in PHD

*PHD* patients treated with hemodialysis, *PPD* patients treated with peritoneal dialysis, *NA* not available, *AE* adverse event, *SAE* serious adverse event

## Efficacy of COVID-19 vaccination in patients treated with hemodialysis

The effectiveness of COVID-19 vaccines is determined primarily by the humoral and cellular immunity produced by the patient in response to the vaccine. Many studies have demonstrated that COVID-19 vaccines produce adequate immune protection in the general population, with protection rates of 74.0–95% [74–76]. However, vaccine-induced immune protection is limited in patients treated with hemodialysis due to immunodeficiency. Many studies have been conducted worldwide to investigate the immune response to COVID-19 vaccines in patients treated with hemodialysis. Goupil and colleagues studied the short-term antibody response in patients treated with hemodialysis after one dose of the BNT162b2. The results showed that most patients treated with hemodialysis who had not been infected with SARS-CoV-2 failed to develop humoral immunity after one dose of BNT162b2 vaccine even after prolonged observation, whereas patients who had previously been infected with SARS-CoV-2 had a slightly better, but delayed response after a single dose of the vaccine. Therefore, the investigators recommended that patients treated with hemodialysis receive two doses of vaccine three administered weeks apart [82]. Jahn et al. compared the humoral immune responses of patients treated with hemodialysis and healthy healthcare workers of all ages after two doses of the BNT162b2 mRNA vaccine. Healthy healthcare workers showed a stronger antibody response, with a median anti-SARS-CoV-2 IgG antibody titer of 800.0 Au/mL, while the antibody response in patients treated with hemodialysis aged under 60 years was similar to those of healthy controls, with a median antibody titer of 597.0 Au/mL (*P* = 0.051). However, the antibody response in patients treated with hemodialysis was negatively correlated with age, resulting in a significantly lower antibody titer of only 280.0 Au/mL in patients treated with hemodialysis aged over 60 years (*P* < 0.0001) [83]. In addition, other studies showed that younger age [84, 85], previous SARS-CoV-2 infection [86] and higher serum albumin levels [87] are positively associated with antibody response, whereas older age [84, 85], longer duration of dialysis [87] and receipt of immunosuppressive therapy [88] were unfavorable factors for antibody response.

The association between humoral and cellular immunity to COVID-19 vaccines in patients treated with hemodialysis is also worth exploring. Gonzalez–Perez et al. investigated the cellular and humoral immune responses to mRNA-1273 vaccination in patients on hemodialysis with or without a previous history of SARS-CoV-2 infection, and compared the results with healthy subjects. Patients on hemodialysis with no prior history of SARS-CoV-2 infection developed effective cellular and humoral immune responses only after the second dose of vaccine, while patients on hemodialysis with prior SARS-CoV-2 infection showed potent and rapid cellular and humoral immune responses after the first dose, and also demonstrating a strong correlation between cellular and humoral immunity in patients treated with hemodialysis [9]. Interestingly, patients treated with hemodialysis have also been shown to produce similar humoral immune responses compared to those with normal kidney function, although patients treated with hemodialysis produce significantly higher cellular immune response [89]. Thus, COVID-19 vaccination has been shown to produce a potent immune response in patients treated with hemodialysis.

Although COVID-19 vaccines have been shown to produce adequate humoral and cellular immunity in patients treated with hemodialysis, the durability of the immune response has become a key concern. Based on data from the general population, the decrease in antibody titers observed around 6 months after the second dose of vaccine raises concerns about possible weakening of immunity [90]. In patients treated with hemodialysis, antibody titers peaked in the third month after two doses of the vaccine and decreased significantly after 6 months [91]. Therefore, it is necessary to monitor the vaccination response in patients and to take measures to strengthen the immune response. More details of the Clinical researches related to the efficacy of COVID-19 vaccination in patients treated with hemodialysis are shown in Table 2.

## Vaccination strategy to strengthen the immune response in patients treated with hemodialysis

### Strengthening immunization

Many studies have demonstrated that the standard two-dose COVID-19 vaccination schedule does not provide adequate immune protection for patients treated with hemodialysis [92–94]. In a prospective observational study of the rationality, safety and efficacy of a third dose of mRNA COVID-19 vaccine in patients treated with maintenance hemodialysis, Espi et al. reported a cumulative incidence rate of COVID-19 within 28 days of 1.98% in those with no prior SARS-CoV-2 infection and no previous COVID-19 vaccination. Furthermore, after the first and second doses of vaccine, the cumulative incidence rate within 28 days fell to 0.65% and 0.25%, respectively, and was significantly lower than before the vaccination [95]. However, the protection rate was still low compared to the general population [74, 76]. Therefore, there is a strong need for immune enhancement strategies to improve humoral and cellular immunity in patients treated with hemodialysis. Good tolerance of the third dose of COVID-19 vaccine in patients treated with hemodialysis has been demonstrated [95]. In a comparison of the humoral and cellular responses of patients treated with hemodialysis after second and third doses of the BNT162b2 mRNA vaccines, Melin et al. found that the serological response rate increased from 88% to 95%, the median SARS-CoV-2-spike IgG antibody titers increased from 606 to 9910 Au/mL, and the T cell response rate increased from 55% to 85% [96]. Therefore, a booster immunization program for patients treated with hemodialysis should be on the agenda, especially for patients who have not been infected with SARS-CoV-2.

### Heterologous vaccination

In addition to booster immunizations to improve immune protection, the use of heterologous vaccines is also a good option. Haase et al. conducted a study among patients treated with hemodialysis comparing the humoral immunity and tolerability of heterologous vaccination with ChAdOx1-S-nCoV-19/BNT162b2 and homologous vaccination with BNT162b2/BNT162b2 or ChAdOx1-S-nCoV-19/ChAdOx1-S-nCoV-19. The results showed that heterologous vaccination induced stronger humoral immunity than homologous vaccination, and also resulted in more adverse reactions, but most were manageable [8]. To avoid recurrence of the previously mentioned rare thromboembolic event caused by ChAdOx1-S-nCoV-19, patients who have received one or two doses of ChAdOx1-S-nCoV-19 vaccine may be given a booster dose of BNT162b2. Heterologous vaccination also elicited stronger immunogenicity than homologous vaccination in the general population, demonstrating the flexibility to select heterologous vaccines for basic and strengthening immunization [97–99].

## Conclusion

Patients treated with hemodialysis are at high risk of SARS-CoV-2 infection and mortality, which has been ameliorated by the advent of COVID-19 vaccines and therapeutic drugs. However, these antivirals are used mainly in mild or moderate patients with high-risk factors for progression to severe disease and not available as pre- or post-exposure prophylaxis for COVID-19. In addition, clinical data on the use of anti-COVID-19 drugs in patients treated with hemodialysis are still limited, and further clinical studies are needed. Therefore, vaccination and infection control measures remain the main ways to prevent SARS-CoV-2 infection in these patients. Fortunately, the COVID-19 vaccine is well-tolerated by patients treated with hemodialysis, but the immunodeficiency in this population causes failure of the vaccine to induce adequate humoral and cellular immunity. Therefore, it is necessary to adopt immunization strategies involving booster or heterologous vaccination and to monitor immunogenicity after vaccination to improve immune protection. Appropriate infection control measures are also necessary. Because of the high risk of infection during dialysis in a hospital setting, home hemodialysis can be adopted to reduce the transmission of infections between healthcare providers and patients. However, this approach still faces many challenges, such as inadequate dialysis equipment technology and lack of patient self-management, so specific plans for home dialysis caregivers as well as patients need to be developed. Patients who elect to undergo dialysis in hospital should take appropriate protective measures, maintain social distancing and regular hand washing and wear a mask correctly to avoid virus transmission.

## Data Availability

Data sharing is not applicable to this article as no data sets were generated or analyzed during the current study.

## Abbreviations

COVID-19: Corona Virus Disease 2019
MERS: Middle East Respiratory Syndrome
SARS: Severe Acute Respiratory Syndrome Coronavirus
VLP: Virus-like particle
IVIG: Intravenous immunoglobulin
mAb: Monoclonal antibody
NTD: N-terminal domain
RBD: Receptor-binding domain
NHC: N-hydroxycytidine
NA: Not available
AE: Adverse event
SAE: Serious adverse event
NCT: National clinical trial
ChiCTR: Chinese clinical trial register
PHD: Patients treated with hemodialysis
PPD: Patients treated with peritoneal dialysis
